# Longitudinal study of *Staphylococcus aureus* colonization and infection in a cohort of swine veterinarians in the United States

**DOI:** 10.1186/s12879-017-2802-1

**Published:** 2017-10-19

**Authors:** Jisun Sun, My Yang, Srinand Sreevatsan, Jeffrey B. Bender, Randall S. Singer, Todd P. Knutson, Douglas G. Marthaler, Peter R. Davies

**Affiliations:** 1 Department of Veterinary Population Medicine, University of Minnesota, 385 ASVM, 1988 Fitch Ave, St. Paul, MN 55108 USA; 2 Department of Veterinary and Biomedical Sciences, University of Minnesota, St. Paul, MN 55108 USA

**Keywords:** Veterinarians, MRSA, Swine, Persistent carriage

## Abstract

**Background:**

People working with pigs are at elevated risk of harboring methicillin resistant *S. aureus* (MRSA) in their nose, which is attributable to occupational exposure to animals harboring livestock adapted *S. aureus*. To obtain insight into the biological nature of occupationally related nasal culture positivity, we conducted a longitudinal study of 66 swine veterinarians in the USA.

**Methods:**

The study cohort resided in 15 US states and worked predominantly with swine. Monthly for 18 months, participants self-collected nasal swabs and completed a survey to report recent exposure to pigs and other animals; the occurrence of work related injuries; and any relevant health events such as skin and soft tissue infections or confirmed staphylococcal infections. Nasal swabs were cultured using selective methods to determine the presence of MRSA and methicillin susceptible *S. aureus* (MSSA), and isolates were characterized by *spa* typing and MLST.

**Results:**

Prevalences of *S. aureus* (64%, monthly range from 58 to 82%) and MRSA (9.5%; monthly range from 6 to15%) were higher than reported for the US population (30% and 1.5% respectively). Predominant *spa* types were t034 (ST398, 37%), t002 (ST5, 17%) and t337 (ST9/ST398 13%), a distribution similar to that found in a concurrent study in pigs in the USA. Veterinarians were classified into three groups: Persistent carriers (PC, 52%), Intermittent carriers (IC, 47%) and Non-carriers (NC, 1%). Persistent carriage of a single *spa* type was observed in 14 (21%) of participants, and paired (first and last) isolates from PC subjects had minor genetic differences. Swabs from PC veterinarians carried higher numbers of *S. aureus*. Among IC veterinarians, culture positivity was significantly associated with recent contact with pigs.

**Conclusions:**

Exposure to pigs did not lead to prolonged colonization in most subjects, and the higher numbers of *S. aureus* in PC subjects suggests that unknown host factors may determine the likelihood of prolonged colonization by *S. aureus* of livestock origin. Exposure to *S. aureus* and persistent colonization of swine veterinarians was common but rarely associated with *S. aureus* disease.

**Electronic supplementary material:**

The online version of this article (10.1186/s12879-017-2802-1) contains supplementary material, which is available to authorized users.

## Background

Working and living in close contact with domestic animals facilitates bidirectional interspecies transmission of microbiota. Concerns about the importance of animals as sources of antibiotic resistant pathogens have been heightened by the unveiling of healthy livestock as reservoirs of methicillin-resistant *Staphylococcus aureus* (MRSA) in many parts of the world [1–7]. While most research has focused on the ST398 lineage of livestock associated MRSA that predominates in Europe, several genotypes of *S. aureus* are adapted to livestock, and their relative prevalence varies geographically and among livestock species [2, 8–10].

In developed countries, approximately 20 to 30% of healthy people harbor *S. aureus* in the nasal cavity, and nasal colonization is associated with elevated risk of clinical infections [11, 12]. The most recent (2003–2004) national data for the USA estimated 28.6% and 1.5% of the population to harbor *S. aureus* and MRSA, respectively, in their nasal cavities [13]. *S. aureus* colonization is heterogeneously distributed across subsets of the population classified as ‘persistent’ (or permanent), ‘intermittent’ or ‘non’ carriers, although the criteria for defining persistent carriage vary [14, 15]. Bacterial, host, microbiome, and other environmental factors may influence the likelihood and duration of nasal colonization of humans with *S. aureus* [16–18], but detailed longitudinal studies of the dynamics of nasal carriage and bacterial genetic diversity are necessary to better understand this phenomenon [14]. Because some lineages of *S. aureus* are known to be host-adapted to particular avian or mammalian species [19, 20], and subtle genomic changes can alter host tropism [21], regular exposure of people to *S. aureus* of animal origin further complicates the poorly understood biology of nasal staphylococcal colonization.

*S. aureus* is considered part of the normal bacterial flora of pigs [22], and people working with live pigs are at elevated risk of being culture positive for *S. aureus* and MRSA. Notably, the predominant genotypes detected in humans with animal contact are typically those present in the animal populations with which they have contact [1, 23–29]. Because *S. aureus* are among the most numerous bacteria in bioaerosols of swine barns [30, 31], discriminating between transient contamination of superficial anatomical sites of people (e.g., upper airways or skin) and sustained colonization is problematic, particularly for workers with regular animal contact. Transient contamination may be the most common outcome in people after short term exposure to MRSA positive swine herds [28, 32, 33]. To date there have only been 2 substantial longitudinal studies of MRSA and *S. aureus* colonization in occupationally exposed swine workers, both in the Netherlands. A study of 110 farm workers sampled 6 times over a year reported that 38% were ‘persistent nasal carriers’ of MRSA, but the possibility of repeated exposure and recontamination could not be eliminated [34]. A study of 137 swine veterinarians sampled 5 times over a 2 year period classified 13% of subjects to be persistently colonized with MRSA based on consistent molecular typing of isolates [24]. The rather different estimates (38% vs 13%) of persistent carriage reported in these 2 studiesmay be an artifact of the different sampling protocols and/or experimental subjects (farmers vs. veterinarians).

Fundamental questions remain about the capacity for *S. aureus* lineages disseminated from animals to colonize and cause disease in humans. Veterinarians are likely more informative subjects than farmers for elucidating long term colonization patterns following interspecies exposure as they typically are exposed to multiple herds rather than a single animal population. The goal of this study was to analyze long term patterns of *S. aureus* (including MRSA) colonization in an intensively sampled cohort of US swine veterinarians.

## Methods

The specific aims of the study were to describe the frequency and duration of positive *S. aureus* and MRSA nasal cultures in a cohort of veterinarians having regular contact with varied populations of commercial swine in the USA, and to characterize the genotypes of the isolates detected. The intensity of sampling (monthly for 18 months) was designed to enable more detailed understanding of *S. aureus* colonization patterns than in previous studies of swine workers.

### Recruitment of study participants

Participants for the study were recruited at the annual meeting of the American Association of Swine Veterinarians (AASV) in Denver, CO in 2012. Eligible veterinarians were members of the AASV who were US residents and typically had regular (i.e., > twice per week) professional contact with pigs. A total of 71 veterinarians provided written consent to be research subjects, of which 68 subsequently participated in sample collection. Two participants withdrew during the course of the study (one due to emigration, one due to leaving swine practice), yielding a final cohort of 66 veterinarians who completed the longitudinal sampling protocol. Participants resided in 15 US states (IA, IL, IN, MI, MN, NE, SD, TX, OK, AL, MO, PA, NC, MD, OH), predominantly in the major swine producing regions of the Midwest and Southeast.

### Sample submission and survey data

Collection materials were mailed to the participants who were given written instructions for self-collection of nasal swabs, as well as an instructional video via YouTube. Starting in July 2012, participants were contacted by monthly email and requested to collect and submit a nasal swab via mail. The email message included a link to a survey using an online tool (http://www.surveymonkey.com) for veterinarians to provide information related to recent pig contact (e.g., time since last pig contact, hours worked in the previous week, number of farms visited in the previous week), and events of physical injury and selected health events (occurrence of skin or soft tissue infections, or confirmed staphylococcal infections) occurring in the month preceding sampling. To encourage compliance, sample collection was conducted at the convenience of the participants, and follow-up emails were sent to non-responders to encourage response rates. To determine quantitative bacteriology of *S. aureus*, a one-time cross-sectional sampling was performed on 41 available subjects who attended the 2014 AASV meeting in Dallas, TX.

### Bacteriology

Samples were refrigerated on arrival at the University of Minnesota, and processed in 3 to 4 batches each month as samples typically were received over a 10–14 day period. For the quantitative bacteriology, all samples were collected and processed as one batch within 24 h of collection.

Isolation of *S. aureus* was performed using the methods described previously [35]. Nasal swabs were double enriched in Mueller-Hinton broth (BBL™, MD, USA) supplemented with NaCl (6.5%) and in Phenol-Red Mannitol broth (BBL™, MD, USA) supplemented with 4μg/ml Oxacillin (Sigma-Aldrich, MO, USA). Broths with a color change were inoculated onto chromogenic agar plate (BBL CHROM agar MRSA, MD, USA) and Factor plate (Veterinary Diagnostic Laboratory, University of Minnesota, MN, USA) to culture MRSA and *S. aureus*, respectively. Two colonies per sample were collected for further characterization. DNA was extracted from colonies with 19.5 μl 10 mM Tris-HCl and 0.5 μl Lysostaphin (both Sigma-Aldrich, MO, USA) at 37 °C for 30 min. PCR was used to detect *mecA* and perform *spa* typing. The primers for *mecA* were [F: 5′ GTA GAA ATG ACT GAA CGT CCG ATA A 3′, R: 5′ CCA ATT CCA CAT TGT TCG GTC TAA 3′], and for *spa* [F: 5′ AGA CGA TCC TTC GGT GAG C 3′, R: 5′ GCT TTT GCA ATG TCA TTT ACT G 3′]. PCR master mix (USB HotStart-IT FideliTaq, affymetrix, CA, USA) was used to amplify the DNA under the following conditions: 95 °C for 2 min, 94 °C for 30s, 55 °C for 30s, 72 °C for 1 min with 30 cycles and 72 °C for 10 min. All PCR products were visualized in 1% agarose gel with SYBR Safe dye in 1X TAE buffer (Tris-Acetate-EDTA, Thermo Fisher Scientific Inc., MA USA) for 40 min at 200 V.

### Quantitative bacteriology

Nasal swabs collected from the 41 subjects were transported in one batch on ice to the laboratory and placed in 1 ml Mueller-Hinton broth tubes within 24 h. Ten-fold dilutions were prepared from 100ul broth (up to 10^−4^) and 100ul from each dilution was spread on a Factor plate and incubated at 37 °C for 22 h. before counting by observers blinded to the carrier status of subjects to determine CFU/swab.

### Molecular typing and analysis

All selected *S. aureus* isolates were subtyped using *spa* typing [36]. After amplification of *spa*, PCR products were cleaned up with Illustra Exoprostar, (GE Healthcare Bio-sciences, PA, USA) and sequenced at the University of Minnesota Genomics Center. Sequences aligned using Sequencher 5.1 software (Gene Codes Corporation, MI, USA) were submitted to the Ridom *spa* typing database (http://spa.ridom.de/index.shtml).

Multi-locus sequence typing (MLST) of *S. aureus* was performed following methods reported previously via the MLST database of *S. aureus* (http://saureus.mlst.net) [37]. MLST typing was performed purposively so that at least one isolate from each *spa* type detected was also evaluated by MLST.

### Definition of carrier status

Consistent with previous studies [14, 15, 24], we classified subjects by carrier status to be non-carriers (NC), intermittent carriers (IC), or persistent carriers (PC). A carrier index (range 0 to 1) was defined as the proportion of sampling events that yielded a *S. aureus* (including MRSA) isolate. Non-carriers were defined as subjects that were never positive for *S. aureus* (including MRSA), and intermittent carriers were culture positive at least once occasion with a carrier index of <0.8. Persistent carriers had a carrier index of 0.8 or greater. The cut-off of 0.8 was based on *post hoc* evaluation of the frequency distribution of the carrier index and was considered conservative (i.e., biased against false positive misclassification of persistent carriage). PC subjects were further classified as true persistent carriers (TPC) if a single *spa* type of *S. aureus* was recovered at all positive sampling events.

### PCR testing for *scn, chp, sak* for the immune evasion cluster (IEC)

A purposively selected subset of 116 isolates was tested for presence of *scn, chp,* and *sak* of the IEC, considered as markers of adaptation to humans. The selection protocol utilized 4 categories of veterinarians 1) TPC subjects colonized with 1 *spa* type; 2) PC subjects colonized with more than 1 *spa* type; 3) IC subjects; 4) IC subjects colonized at least once with MRSA. For TPC subjects (category 1), the first and last isolates obtained were selected for each veterinarian. For category 2 subjects in whom the detected genotype changed over time, 2 isolates from each *spa* type were chosen up to 4 isolates per veterinarian (i.e., 2 predominant *spa* types). For category 3 subjects, only the predominant *spa* type was selected. Both MRSA and MSSA were detected from 4 IC subjects during the study period (category 4), and one MRSA isolate and an isolate of the predominant MSSA *spa* type were selected. Thus, a total of 24 MRSA and 92 MSSA were tested for *scn, chp, sak* following the methods previously described [38]. Annealing temperatures for the *scn, chp* and *sak* were 63 °C, 51.5 °C and 53 °C, respectively. All PCR products were electrophoresed in 1% agarose gel stained with SYBR Safe dye in 1X TAE buffer (Tris-Acetate-EDTA, Thermo Fisher Scientific Inc., MA USA) for 40 min at 200 V and visualized on a UV transilluminator. ATCC 700698 (Mu3), ATCC 700699 (Mu50) and ATCC 25904 (Newman) were used as positive controls for *scn, chp* and *sak*, respectively.

### Whole genome sequencing

Eighteen isolates were selected from 9 persistent carriers (three subjects per sequence type) to evaluate genomic variation across the study period. Isolates from the first (month 1) and last (mostly month 18) samples collected for each subject were included. One veterinarian was colonized with ST5-t062 for 11 months, then ST398-t011 *S. aureus* for the remaining 7 months. For this subject, ST5-t062 isolates from month1 and month11 were selected. Another veterinarian was colonized with ST398-t034 MRSA followed by a closely related *spa* type (ST398-t011 MRSA) for the final 4 months. Genomic DNA was extracted from overnight cultures in LB (Lysogeny broth, BD Difco™, NJ, USA) using the Qiagen Blood and Tissue Kit (Valencia, CA, USA) following the manufacturer’s instructions. Approximately 10 ng of extracted DNA per sample was sent to University of Minnesota Genomics Center (Minneapolis, MN, USA). Independent next generation sequence (NGS) libraries (Nextera DNA Library Preparation Kit, CA, USA) were created for each sample, pooled onto a single lane HiSeq 2500 rapid-run, and 250 bp paired-end reads were generated. Runs yielded an average of 2.3 million reads per sample, and 82% of reads had a quality score (Phred + 33) greater than 30 (details described in Additional file 1). To estimate genetic distances, the isolates were aligned using Mauve (ver.2) and single nucleotide polymorphisms (SNPs) from aligned genomes were extracted and imported into MEGA 7.0 software to generate a maximum likelihood phylogenetic tree employing 100 bootstrap iterations.

### Comparison with *S. aureus* isolates from veterinarians and from pigs in the USA

The prevalence and genotypic characterization of a geographically diverse sample of *S. aureus* collected from pigs in the USA was recently published [8]. The majority (36 of 38) of farms included in that study were served by 36 veterinary participants of the current study, providing a congruent time-space window to underpin the comparison of genotypes detected in US pigs and swine veterinarians. *Spa* types from the current study and from the study of pigs were categorized to be ‘shared’ (if detected in both species), ‘swine only’ or ‘human only’. A minimal spanning tree (MST) for clustering of *spa* types was constructed using the Bionumerics 7.1 (Applied Maths, SintMartens-Latem, Belgium).

### Statistical analysis

Univariate analysis was performed to evaluate associations between culture positivity of *S. aureus* and working activities related with occupational exposure using R studio (Version 0.99.892). The exposures related to animal contact were: last contact with pigs (categorical variable, 0: same day; 1: previous day, 2: 2 days previously, 3: 3 days, 4: more than 3 days); hours of pig contact (continuous); and the number of farms visited in the previous week (continuous). As these self-reported exposure variables were correlated, multivariable analysis was limited to last contact with pigs, which was considered the most reliable and relevant variable with respect to transient contamination. To account for repeated observations on the same subjects over time, a two-way nested mixed-effects model was used, with samples nested within veterinarians, and the fixed effect of time since last contact with pigs (Days: same day vs. more than 1 day). The model was performed in R software environment 3.0, via lme4 package as follows:

$$ \mathrm{Logit}\ \left({\mathrm{p}}_{\mathrm{i}}\right)=\mathrm{Intercept}+{\mathrm{Days}}_{\mathrm{i}}+{\mathrm{Vet}}_{\mathrm{i}}+{\mathrm{Sample}}_{\mathrm{i}\mathrm{j}} $$

In addition, wearing a facemask at last pig contact, and occurrence of injuries by livestock or soft tissue infections were also evaluated. The numbers log_10_CFU (colony forming unit) of putative *S. aureus* colonies per swab was compared between intermittent carriers and persistent carriers using Mann-Whitney U test.

## Results

Two veterinarians withdrew from the study due to emigration or altered work circumstances, leaving a cohort of 66 swine veterinarians who provided monthly nasal swabs. One veterinarian stopped working with swine after 7 months, but completed the sampling protocol and was retained in the study. Compliance with swab submission and survey completion was over 99% for both nasal swab submission (1179/1188) and survey submission (1177/1188). The median interval between sample collection and sample processing was 4 days (IQ range: 3–5 days; range: 1-35 days) and was not associated with the likelihood of culture positivity (*p* = 0.99).

**Fig. 1:**
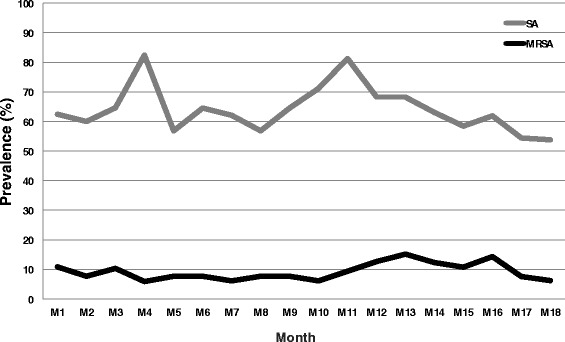
Proportion of *S. aureus* and MRSA positive nasal swabs from a cohort of swine veterinarians sampled monthly from July 2012 to December 2013

Overall, *S. aureus* was detected in 63.7% (757/1188) of monthly nasal swab samples (yielding 1356 *S. aureus* isolates characterized) and MRSA in 9.5% (113/1188) samples (yielding 213 MRSA isolates characterized). The monthly apparent prevalence of *S. aureus* ranged from 58% to 82%, while apparent prevalence of MRSA ranged from 6 to 15%, and there was no indication of seasonal or longer term trends in prevalence over the course of the study (Fig. 1). MRSA was detected at least once during the study in 18 (27%) of subjects.

**Fig. 2:**
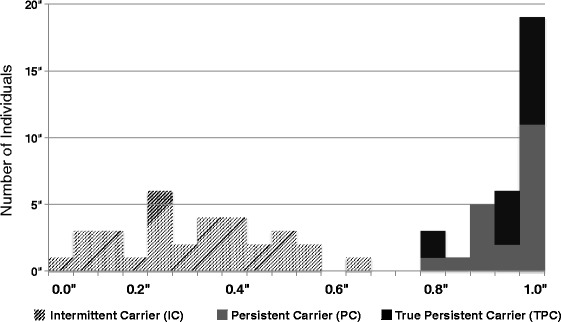
Histogram of the proportion of sampling events yielding a *S. aureus* isolate (“Carrier index”)

At the individual level, the proportion of positive sampling events ranged from 0% (one veterinarian) to 100% (18 veterinarians) and was clearly bimodally distributed (Fig. 2). In univariate analyses, the likelihood of a culture positive result was negatively associated with the interval between the last contact with pigs and collection of the sample (*P* = 0.001), and positively associated with the hours of pig contact per week (*P* = 0.02), but not associated with the number of farms visited (*P* = 0.09) in the previous week. Multivariable analysis to adjust for repeated observations on the same veterinarians showed the odds or a positive culture were reduced by 33% for samples collected one day or longer after pig contact (OR = 0.67; 95% CI: 0.46–0.96) compared with same day collection. Three subjects reported having a staphylococcal infection during the study (2 MSSA, 1 MRSA), all of which were described as localized and did not lead to hospitalization or time off work. This corresponds with an incidence of 2.5 cases per 1000 person months.

Of the 66 subjects, 31 (47%) were classified as intermittent carriers and 34 (52%) were classified as persistent carriers of *S. aureus*. Based on consistent detection of a specific *spa* type at all culture positive samplings, 14 (21%) veterinarians were classified as true persistent carriers (TPC). The TPC group included the veterinarian who ceased working with pigs after 7 months but remained positive with ST398/t034 (methicillin susceptible) for the remaining 10 months. The majority (60%) of MRSA isolations were from 4 PC subjects who were positive for MRSA (all ST398) on at least 15 occasions. One of the PC MRSA subjects, who consistently harbored ST398/t034 MRSA, worked exclusively in one production system where this *spa* type occurs at high prevalence, and which was used as a MRSA positive control farm in the related swine study [8].

### Whole genome sequencing

**Fig. 3:**
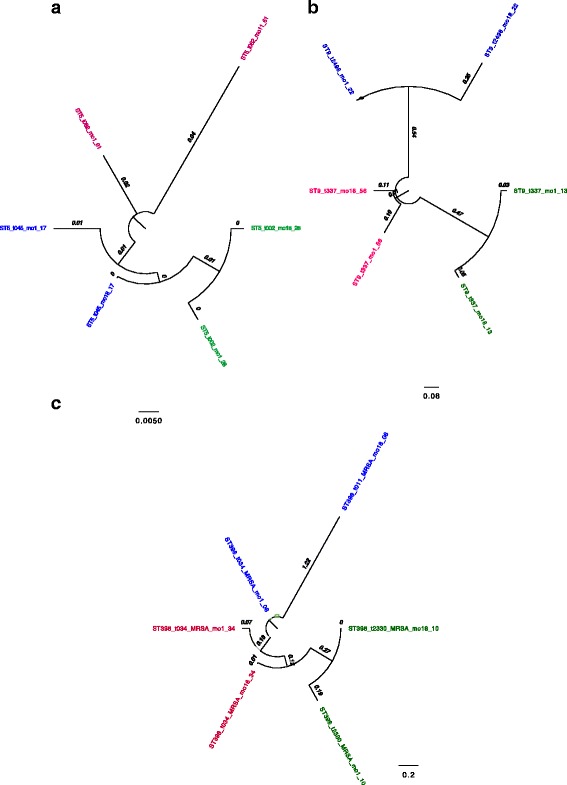
Genomic distance between isolates from beginning of study and the end of study in persistent carriers displayed by Sequence Type (**a**) ST5, **b**) ST9, **c**) ST398). Within sequence type, the isolates from same person were colored with same color. Names of each isolate were described by ST_spa type_ (if MRSA)_sampling month_vetID. The bar scales on each ST indicate number of nucleotide difference per site

Comparison of the isolates from 9PC subjects (3 each of sequence types ST398, ST9 and ST5) generally indicated close genetic similarity between the first and last isolates collected. All paired isolates within subjects had 0.01 and 0.2 nucleotide differences per site within ST5 and ST398 respectively (Fig. 3). Pairs of isolates of the same genotype (ST9-t337) showed greater similarity within subjects than between subjects (Fig. 3b).

Overall, the *S. aureus* isolates were distributed among 27 *spa* types within 8 MLST sequence types (Table 1). Three sequence types (ST398, ST5, ST9) constituted over 94% of the *S. aureus* isolates. Over the course of the study, ST398 isolates were detected at least once in 63 (83%) veterinarians; ST5 isolates in 43 (65%) veterinarians, ST9 in 29 (56%) veterinarians; and other MLST types in 8 (12%) veterinarians. The 3 predominant MLST types (ST398, ST5, ST9) were all isolated at least once from 23 (35%) subjects. Within each of these 3 MLST types, a single *spa* type (t034, t002, and t337, respectively) constituted approximately 70% of isolates. Two of these sequence types, ST398 (80.8%) and ST5 (14.1%), accounted for almost 95% of all MRSA isolates, with remainder being ST8/t008, a common human MRSA variant considered unlikely to be of swine origin.

**Table 1:** Numbers (%) of *spa* types of *S. aureus* and MRSA isolated from swine veterinarians, by MLST type

Sequence type	*Spa* type	*S. aureus* (*n* = 1356)	MRSA (*n* = 213)
ST398	t034	462 (34.1)	116 (54.5)
	t571	71 (5.2)	0
	t011	63 (4.6)	12 (5.6)
	t337^a^	20 (1.5)	0
	t3446^a^	19 (1.4)	0
	t1250	8 (0.6)	0
	t2330	6 (0.4)	44 (20.7)
	t2876	24 (1.8)	0
	t7160	13 (1.0)	0
	t1255	3 (0.2)	0
		650 (51.8)	172 (80.8)
ST5	t002	238 (17.6)	27 (12.7)
	t045	69 (5.1)	0
	t062	19 (1.4)	0
	t242	0	3 (1.4)
	t570	12 (0.9)	0
	t856	3 (0.2)	0
		341 (25.1)	30 (14.1)
ST9	t337^a^	178 (13.1)	0
	t2498	47 (3.5)	0
	t10494	11 (0.8)	0
	t3446^a^	9 (0.7)	0
	t1334	3 (0.2)	0
	t1430	2 (0.1)	0
		289 (18.4)	0
ST8	t008	0	11 (5.1)
	t2196	18 (1.3)	0
ST30	t338	4 (0.3)	0
	t363	1 (0.1)	0
ST72	t126	30 (2.2)	0
ST278	t330	22 (1.6)	0
ST2007	t8314	1 (0.1)	0

^a^Two sequence types were identified within t337 and t3446 isolates

**Fig. 4:**
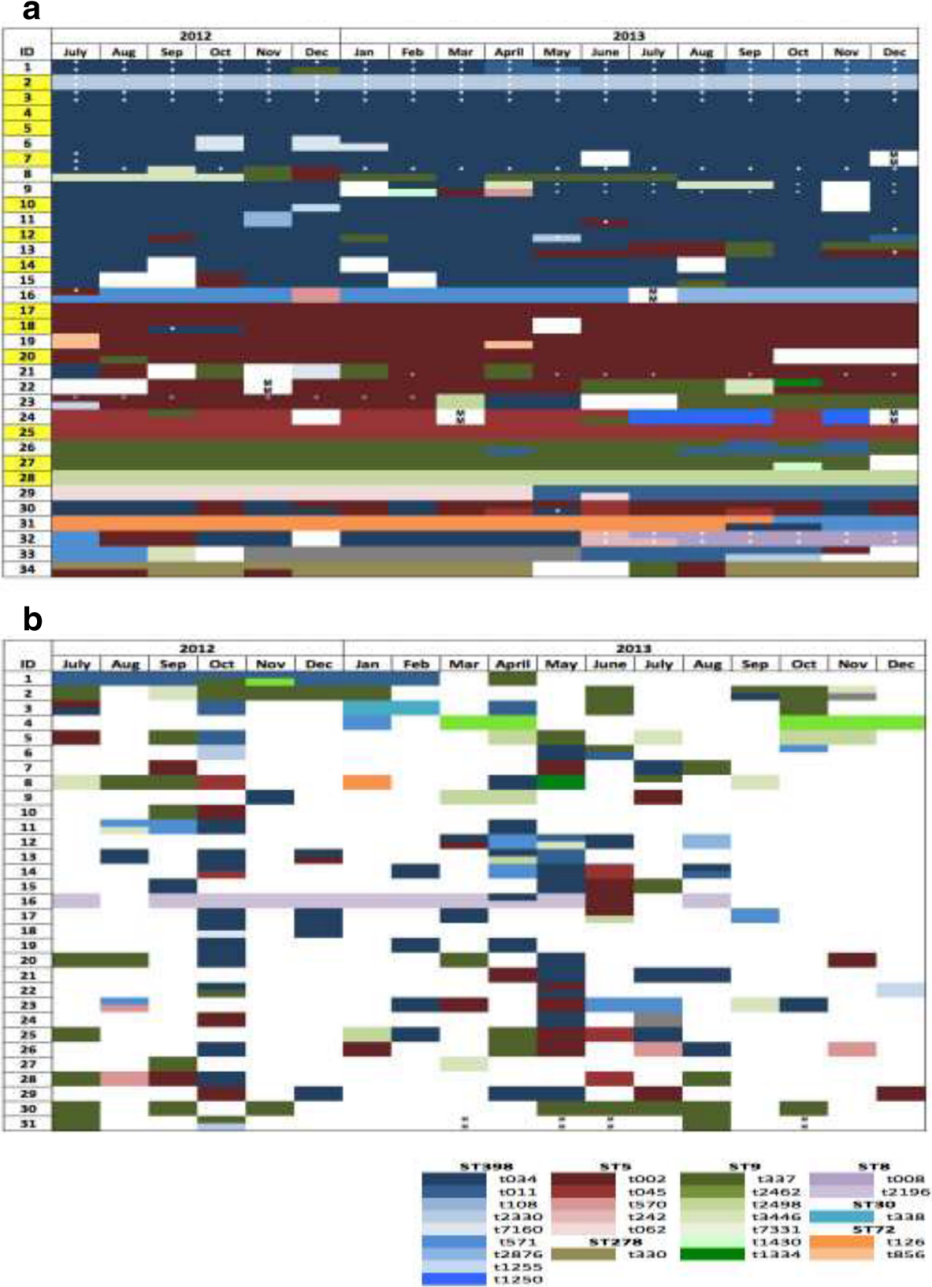
Patterns of detection of *S. aureus spa* types in veterinarians categorized as permanent carriers (**a**) and intermittent carriers (**b**). Missing samples are indicated as ‘M’, white spaces indicate culture negative events; white dots signify methicillin resistant isolates (typically 2 isolates typed per month). Yellow boxes in (**a**) indicate true persistent carriers. Colors also reflect the MSLT type of the major sequence types being ST398 (blue shades), ST5 (red shades), and ST9 (green shades)

The detection patterns of specific *spa* types over time varied enormously from highly consistent presence of individual *spa* types in TPC subjects, to very inconsistent patterns with multiple *spa* types detected in individual veterinarians over time (Fig. 4a, b). More than one *spa* type was detected in 71 (6%) of the monthly nasal swab samples. It is also notable, that in some of the PC and IC subjects a single *spa* type was detected over multiple consecutive months, but thereafter other *spa* types were detected.

In the quantitative comparison of PC and IC veterinarians among 41 AASV members sampled in 2014, PC veterinarians (18 of 22; 82%) were more likely to be nasal culture positive than IC (4 of 19, 21%). Culture positive swabs from PC subjects harbored approximately 2 logs more *S. aureus* per swab than positive swabs from IC subjects (*P* = 0.05). *Spa* typing of isolates from this non-selective procedure yielded the same spa types found at other months for each TPC subject, and the same spa type detected in the most recent positive culture for 2 PC subjects.

## IEC genes testing

Approximately 10% (one MRSA and 11 MSSA) of the 116 tested isolates were positive for two or three of the IEC genes (Table 2). A single isolate (t2196, ST8) was only positive for the *scn* and *sak* genes. The majority of isolates positive for IEC genes were *spa* types likely to be of human origin apart from the t011 (ST398), t5883 (ST398) and t002 (ST5) isolates.

**Table 2:** *S. aureus* isolates testing positive for IEC

	Month^a^	ID	MRSA	*spa* type (ST)	*scn*	*sak*	*chp*
1	04	24	MSSA	t126 (ST72)	+	+	+
2	04	57	MSSA	t330 (ST278)	+	+	+
3	04	61	MSSA	t062 (ST5)	+	+	+
4	06	19	MSSA	t5883 (ST398)	+	+	+
5	07	05	MSSA	t338 (ST30)	+	+	+
6	09	44	MSSA	t2196 (ST8)	+	+	–
7	12	61	MSSA	t062 (ST5)	+	+	+
8	12	61	MSSA	t011 (ST398)	+	+	+
9	16	57	MSSA	t330 (ST278)	+	+	+
10	17	41	MRSA	t008 (ST8)	+	+	+
11	17	66	MSSA	t002 (ST5)	+	+	+
12	18	66	MSSA	t002 (ST5)	+	+	+

^a^Month of sampling from month 1 to month 18

## Comparison with *S. aureus* isolates from veterinarians and from pigs in the USA

**Fig. 5:**
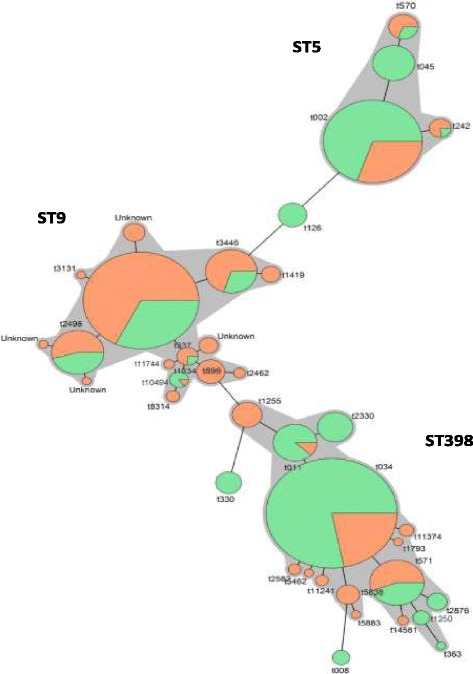
Genetic relatedness of *S. aureus* isolates from swine (*n* = 1193) and veterinarians (*n* = 1659). Each node in this minimum spanning tree depicts one of 38 *spa* types identified from swine and swine veterinarians. The size of circles denotes the number of isolates. Swine isolates and vet isolates are color coded with orange and green, respectively

*Spa* types from the current study and a previous pig study [8] were categorized as ‘shared’ if detected in both species, ‘swine only’ or ‘human only’. Thirteen *spa* types were shared and accounted for 83% and 92% of all isolates from veterinarians and pigs, respectively. Twenty *spa* types were found only among swine isolates while 14 *spa* types were identified only among veterinary isolates (Table 3). A minimal spanning tree (MST) analysis for clustering of *spa* types from the swine and veterinary studies was constructed (Fig. 5). As expected, isolates were clustered together by sequence type. ST9 isolates were more likely to be found among pig isolates while *spa* types belonging to ST398 and ST5 were relatively more frequent among isolates from veterinarians.

**Table 3:** *Spa* type comparison between swine and veterinary isolates

Shared	Swine only	Human only
t002	t899	t008
t011	t5883	t045
t034	t5838	t062
t10494	t5462	t1250
t1255	t3232	t126
t1334	t306	t1430
t242	t2582	t2196
t2498	t2462	t2330
t337	t2315	t2876
t3446	t1793	t330
t570	t14851	t338
t571	t1419	t363
t8314	t11744	t856
	t11374	t922
	t11241	
	unknown1	
	unknown2	
	unknown3	
	unknown4	
	unknown5	

*Repeat succession of unknown types: Unknown1 (r07r16r23r23r02r12r17r23r02r34), Unknown2 (r07r16r16r16r23r23r02r12r23r02r34), Unknown3 (r07r16r16r23r02r12r23r02r34), Unknown4 (r08r475r2r25r2r25r34r34r25), Unknown5 (r07r16r23r23r02r23r02r34)

## Discussion

The high prevalence of *S. aureus* recovered from nasal cultures reflects the increased exposure to *S. aureus* occurring in livestock environments [4, 26, 39]. Although some influence of methodological differences cannot be eliminated, the overall *S. aureus* prevalence (64%) in swine veterinarians is approximately double that estimated in studies of the overall US population [13] and in other developed countries [11, 12]. However, it is very similar to a 72% prevalence in a study of Dutch swine veterinarians [24]. The prevalence of MRSA (9.5%) was also higher than reported in the US population (1.5%), but was substantially lower than in the Dutch swine veterinarians (44%), which likely reflects the lower prevalence of MRSA in the US swine industry relative to the Netherlands [4, 8, 40].

A substantial majority (>84%) of *S. aureus* isolates in this study were deemed likely to be of swine origin based on several criteria. A parallel study of 38 US swine farms, sampled by a subset of the veterinarians in the current study, found that three MLST sequence types (ST9, ST398, ST5) constituted over 99% of swine isolates, with *spa* types t337, t034, and t002 predominating [8]. The same sequence types and *spa* types were similarly predominant among the veterinarians, generally lacked the IEC genes, and were predominantly (85%) tetracycline resistant [41]. The absence of the IEC genes, together with tetracycline resistance, has been used previously to differentiate isolates of human and animal origin [42, 43] and our data suggest that *S. aureus* acquired from swine may largely displace *S. aureus* of human origin in the nasal flora of swine veterinarians. However, given that more than one *spa* type was often detected in individual samples, and only 2 isolates per sample were categorized, it is possible that carriage of relatively low numbers of human *S. aureus* by veterinarians went undetected [44], and that animal contact added to, rather than displaced human *S. aureus*. Regardless, the data suggest that animal exposure alters the composition of the nasal *S. aureus* populations of swine workers. Any resultant impact on risk of clinical infection will depend on the relative persistence, transmissibility, and pathogenicity of *S. aureus* of swine origin compared with human adapted variants.

The primary goal of this study was to understand the persistence of *S. aureus* of swine origin in occupational groups that are in close contact with pigs. Several previous studies of livestock workers have examined this question, with varying outcomes [3–5, 26, 29, 32, 39]. Most studies focused on MRSA alone, and the frequency and duration of sampling has varied widely. A common obstacle to inference has been the inability to differentiate repeated contamination of the nasal mucosa from true persistent colonization [45], particularly for farmers who are repeatedly exposed to the same herd (and *S. aureus* populations) [39]. We specifically studied veterinarians because they generally visit multiple farms and therefore should be exposed to more diverse *S. aureus* populations.

Longitudinal studies of nasal carriage of *S. aureus* in humans typically classify subjects as persistent carriers, intermittent carriers, and non-carriers [14, 46]. It is believed that a subset (usually of the order of 20%) of healthy people are persistently colonized with *S. aureus* [15, 47], and this is associated with higher risk of *S. aureus* clinical infections [48, 49]. Both host genetic factors and microbial factors may play a role in determining duration of carriage [16], and it was recently reported that presence of *S. lugdunensis* in the nose may suppress *S. aureus* populations [18]. Currently, there is no accepted consensus for defining persistent carriers, and categorization of individuals will be influenced by study design (particularly the frequency and duration of sampling), and the cut-off (carrier index) used to define carriage status [46]. We employed a *post-hoc* epidemiological approach to establish a cut-off (carriage index >0.8) to define persistent carriage. The same criterion has been employed in previous studies [14, 46]. The bimodal distribution of the frequency of culture positive samples among veterinarians (Fig. 2) suggests that even in environments with high exposure to *S. aureus* of animal origin, individual host characteristics are likely important determinants of the persistence of colonization. This inference is further supported by the substantially higher numbers of *S. aureus* recovered from nasal swabs from PC compared with IC veterinarians, which is consistent with quantitative studies performed in humans both with [39] and without [50] known livestock association.

The proportion of persistent carriers (52%, 34/66) was considerably higher, and the proportion of non-carriers (<2%, 1/66) considerably lower, than reported in people without livestock contact [14, 46, 51]. This may reflect unusually frequent exposure to *S. aureus* that can occur in intensive swine facilities [52]. Very similar rates of apparent persistent carriage of *S. aureus* were also observed in recent longitudinal studies of swine farmers (52%, [39]) and swine veterinarians (47%, [24]) in the Netherlands, although the sampling protocols and criteria for defining persistence in those studies differed.

The associations observed between culture positivity and time since last pig contact and hours of pig contact in the prior week are consistent with previous studies indicating that occupational exposures to animals often result in transient contamination [28, 32, 53]. Collection of swabs on the same day following pig contact substantially increases the odds of a positive culture, therefore sampling schedules will influence estimates of prevalence. The variability of *spa* types detected over time in the IC group (Fig. 4b) also attests to multiple exposures of swine veterinarians to diverse *S. aureus* populations. Although early research of persistent carriage in humans suggested that people were colonized over the long term by a single *S. aureus* variant, more recent studies indicate that *S. aureus* variants are often replaced over time [14, 46, 51]. Ritchie (2015) sampled 122 healthy young adults weekly over 13 weeks and described 3 patterns of persistent carriage, being continuous carriage of a single *spa* type; an abrupt change from one *spa* type to another; and periods of co-carriage with two *spa* types. Although ‘strain turnover’ was observed in both intermittent and persistent carriers in that study, the majority (63%) of PC subjects were colonized by a single *spa* type. We made similar observations in the veterinary cohort where all 3 patterns of PC were observed, and a substantial proportion (41%; 14 of 34 PC) of the PC veterinarians were classified as ‘true persistent colonization’ due to the repeated presence of a single *spa* type over 18 months. This slightly lower proportion (41% vs. 63%) of TPC could be an artifact of the longer sampling period (18 months vs. 13 weeks) providing more opportunity for strain turnover, or the additional exposure to *S. aureus* variants that occurs in the livestock environment. Our observations with WGS provided further evidence that true persistent colonization with animal origin of *S. aureus* can occur in some subjects. The paired isolates from each persistent carrier (18 months apart) had shorter genomic distances than seen between isolates of same *spa* type but from different individuals. A similar inference was reached in a previous study using whole genome mapping of isolates from 16 Dutch veterinarians [54]. Although it is not possible to eliminate the possibility of repeated reacquisition of the same variant from pigs by veterinarians, this is considered unlikely over an extended period unless they were exposed to homogeneous swine populations harboring few *S. aureus* variants. However, repeated reacquisition from pigs can be eliminated for one subject in our study who remained culture positive for ST398-t034 MSSA for 9 months after leaving swine practice. Current evidence suggests that a substantial proportion of swine veterinarians become colonized with *S. aureus* of swine origin for at least 18 months to 2 years [24], and can harbor substantial numbers of these organisms. Furthermore, although several studies indicate that ST398 MRSA are less transmissible among people than MRSA of human origin, transmission from veterinarians to their families, who also may become persistently infected, has been clearly demonstrated [54, 55]. Also, persistent colonization of humans with swine *S. aureus* isolates may be a mechanism for transmission among herds. This is arguably inconsequential given that *S. aureus* is part of the normal commensal flora of swine, but could be problematic for efforts to prevent MRSA transmission among herds [56].

The fact that some swine workers harbor substantial numbers of *S. aureus* with genotypes consistent with swine origin has implications regarding occupational health. However, the human health consequences of livestock associated *S. aureus* are not well defined, and available information is largely limited to ST398 MRSA. In the USA, to date *S. aureus* of animal origin appear to have had negligible impact on human health. The reported incidence of *S. aureus* infections in this study (2.5 per 1000 person months, or 3% per veterinary-year) is similar to that reported in a study of Iowa residents (2.7 per 1000 person months). Furthermore, *spa* types linked to livestock represented only 1% of *S. aureus* isolates, and 0.24% of MRSA isolates, from human clinical infections in the pig dense state of Iowa [57]. In contrast, a substantial study in Denmark showed increased likelihood of infection with specific livestock associated MRSA, but not increased MRSA infection risk overall, in people living in pig dense areas [58]. Transmission from swine workers into the broader community is a plausible explanation. However, despite the substantial exposure to *S. aureus* of swine origin that occurs in intensive production environments, there are remarkably few reports of medically significant infections occurring in swine workers [45, 59]. To date, we are unaware of any studies demonstrating increased risk of clinical *S. aureus* infections in livestock workers. A recent prospective study of swine farm workers in Holland found that carriage of ST398 MRSA was not associated with elevated risk of infections, healthcare contact, or measures of reduced quality of life [60]. Somewhat surprisingly, the small numbers of fatal human cases of ST398 infection have occurred in people without known livestock contact, but who were generally medically compromised [61]. Even though there are a small number of fatal human cases of ST398 infections, it is important to better understand the interchange of *S. aureus* between humans and animals and the implications for the transfer of resistance elements.

## Conclusions

Swine veterinarians are continually exposed to *S. aureus* of swine origin when working with pigs. This longitudinal study confirmed that the outcomes of this occupational exposure range from short term contamination of the nasal cavities (reflected in higher prevalence of detection in samples collected soon after animal exposure) to apparent long term colonization (at least 18 months) in some individuals in whom the same spa type was detected repeatedly throughout the study. Exposure to pigs did not lead to prolonged colonization in most subjects, and the higher numbers of *S. aureus *in PC subjects suggests that unknown host factors may determine the likelihood of prolonged colonization by *S. aureus * of livestock origin. Although exposure to *S. aureus* and persistent colonization of swine veterinarians was common, the few reports of clinical *S. aureus* infections reported were of minimal medical significance.

## Additional file

Additional file 1: Supplement methods for whole genome sequencing analysis. (DOCX 108 kb)

## Abbreviations

AASV: American Association of Swine Veterinarians
AL: Alabama
ATCC: American Type Culture Collection
CFU: Colony forming unit
CO: Colorado
IA: Iowa
IC: Intermittent carrier
IEC: Immune evasion cluster
IL: Illinois
IN: Indiana
IQ: Interquartile
MD: Maryland
MI: Michigan
MLST: Multilocus sequence typing
MN: Minnesota
MO: Missouri
MRSA: Methicillin resistant *Staphylococcus aureus*
MSSA: Methicillin susceptible *Staphylococcus aureus*
MST: Minimal spanning tree
NC: Non-carrier
NC: North Carolina
NE: Nebraska
OH: Ohio
OK: Oklahoma
PA: Pennsylvania
PC: Persistent carrier
PCR: Polymerase chain reaction
*S. aureus*: *Staphylococcus aureus*
SD: South Dakota
SNPs: Single nucleotide polymorphisms
spa: *Staphylococcus aureus* protein A
ST: Sequence type
TPC: True persistent carrier
TX: Texas
