# Supplementary material for: Longitudinal study of Staphylococcus aureus colonization and infection in a cohort of swine veterinarians in the United States
Source: BMC Infect Dis. 2017 Oct 19;17:690. doi: 10.1186/s12879-017-2802-1 (PMC5649086; doi:10.1186/s12879-017-2802-1)
Supplement: Additional file 1: — Supplement methods for whole genome sequencing analysis. (DOCX 108 kb) [file 12879_2017_2802_MOESM1_ESM.docx]

**Supplement methods for whole genome sequencing analysis**

**Next generation sequencing**

Genomic DNA was extracted from overnight cultures in LB (Lysogeny broth, BD Difco™, NJ, USA) using the Qiagen Blood and Tissue Kit (Valencia, CA, USA) following the manufacturer’s instructions. Approximately 10ng of extracted DNA per sample was sent to University of Minnesota Genomics Center (Minneapolis, MN, USA). Independent NGS libraries (Nextera DNA Library Preparation Kit, CA, USA) were created for each sample, pooled onto a single lane HiSeq 2500 rapid-run, and 250 bp paired-end reads were generated. The run yielded an average of 2.3 million reads per sample. 82% of reads had a quality score (Phred+33) greater than 30 (mean percentile across all samples).

**Read-set filtering**

Raw reads were de-multiplexed and quality control metrics were determined using FastQC (v0.11.2) software (<http://www.bioinformatics.babraham.ac.uk/projects/fastqc/>.). All reads were quality filtered and adapter sequences were trimmed using Trimmomatic(v0.33) (Bolger et al. 2014) . The minimum length was set to 125 bp. All reads that contained greater than 70% of any single nucleotide were removed by Prinseq (v0.20.4) (Schmieder and Edwards, 2011). Despite sequencing pure bacterial isolates, we further identified and removed any non-*Staphylococcus aureus* (chromosome or plasmid) reads from our filtered read-set using Kraken software (v0.10.5-beta) (Wood and Salzberg, 2014) with a custom database containing 33,778 bacterial strains (complete genomes, chromosomes, or scaffolds) from the RefSeq database (<http://www.ncbi.nlm.nih.gov/books/NBK21101>).

**Genome Assembly**

*De novo* genome assembly of our filtered read-set was completed using Megahit (v1.0.4-beta-3-g027c6b6) (Li et al. 2015; Li et al. 2016) with the following assembly options: --no-mercy --min-count 3 --k-min 105 --k-max 225 --k-step 10 --prune-level 3. Initial assemblies were analyzed using QUAST (v3.1) (Gurevich et al. 2013) revealing that the mean number of contigs assembled was 248 (80 min, 903 max) per sample containing a mean of 2,728,240 bp (2,678,477 min, 2,789,826 max) and a mean N50 value of 41,539 bp (4,787 min, 124,222 max). These draft assemblies were improved by remapping all trimmed reads to the assembly using Bowtie 2 (v2.2.4) (Langmead and Salzberg, 2012), bam files were sorted in samtools(v1.2) (Li et al. 2009; Li. 2011), and Pilon (Walker et al. 2014) (v1.16) was used to correct regions of mis-assembly using default settings. Contigs were extended using SSPACE (v3.0) (Boetzer et al. 2011) and the closest reference genome for each sample was discovered by BLASTn comparison against a database of 215 *Staphylococcus aureus* complete chromosomes and 673 plasmids from GenBank, using NCBI_blast+ (v2.2.28) (Camacho et al. 2009). For each isolate, contigs were aligned to its closest reference strain using CAR (Lu et al. 2014) (version July 14, 2014) and MUMer (v3.23) (Kurtz et al. 2004) software, which positioned and orientated contigs into a single scaffold, and gaps between contigs were connected with ‘N’s. Gap-lengths were reduced using Gapfiller(v1.10) (Boetzer and Pirovano, 2012) and finally SNPs and indels in the draft assembly were corrected by running six iterations of ICORN2(v0.95) software (Otto et al. 2010). Across all samples, mean read coverage was 286x when remapping trimmed reads to completed assemblies. Finally, the completed chromosome and plasmid sequences were annotated with gene and protein information using Prokka (v1.11) (Seemann. 2014).

References

Boetzer, M., Pirovano, W., 2012. Toward almost closed genomes with GapFiller. Genome Biol. 13, R56-2012-13-6-r56.

Boetzer, M., Henkel, C.V., Jansen, H.J., Butler, D., Pirovano, W., 2011. Scaffolding pre-assembled contigs using SSPACE. Bioinformatics 27, 578-579.

Bolger, A.M., Lohse, M., Usadel, B., 2014. Trimmomatic: a flexible trimmer for Illumina sequence data. Bioinformatics 30, 2114-2120.

Camacho, C., Coulouris, G., Avagyan, V., Ma, N., Papadopoulos, J., Bealer, K., Madden, T.L., 2009. BLAST+: architecture and applications. BMC Bioinformatics 10, 421-2105-10-421.

Gurevich, A., Saveliev, V., Vyahhi, N., Tesler, G., 2013. QUAST: quality assessment tool for genome assemblies. Bioinformatics 29, 1072-1075.

Kurtz, S., Phillippy, A., Delcher, A.L., Smoot, M., Shumway, M., Antonescu, C., Salzberg, S.L., 2004. Versatile and open software for comparing large genomes. Genome Biol. 5, R12.

Langmead, B., Salzberg, S.L., 2012. Fast gapped-read alignment with Bowtie 2. Nat. Methods 9, 357-359.

Li, D., Liu, C.M., Luo, R., Sadakane, K., Lam, T.W., 2015. MEGAHIT: an ultra-fast single-node solution for large and complex metagenomics assembly via succinct de Bruijn graph. Bioinformatics 31, 1674-1676.

Li, D., Luo, R., Liu, C.M., Leung, C.M., Ting, H.F., Sadakane, K., Yamashita, H., Lam, T.W., 2016. MEGAHIT v1.0: A fast and scalable metagenome assembler driven by advanced methodologies and community practices. Methods 102, 3-11.

Li, H., 2011. A statistical framework for SNP calling, mutation discovery, association mapping and population genetical parameter estimation from sequencing data. Bioinformatics 27, 2987-2993.

Li, H., Handsaker, B., Wysoker, A., Fennell, T., Ruan, J., Homer, N., Marth, G., Abecasis, G., Durbin, R., 1000 Genome Project Data Processing Subgroup, 2009. The Sequence Alignment/Map format and SAMtools. Bioinformatics 25, 2078-2079.

Lu, C.L., Chen, K.T., Huang, S.Y., Chiu, H.T., 2014. CAR: contig assembly of prokaryotic draft genomes using rearrangements. BMC Bioinformatics 15, 381-014-0381-3.

Otto, T.D., Sanders, M., Berriman, M., Newbold, C., 2010. Iterative Correction of Reference Nucleotides (iCORN) using second generation sequencing technology. Bioinformatics 26, 1704-1707.

Schmieder, R., Edwards, R., 2011. Quality control and preprocessing of metagenomic datasets. Bioinformatics 27, 863-864.

Seemann, T., 2014. Prokka: rapid prokaryotic genome annotation. Bioinformatics 30, 2068-2069.

Walker, B.J., Abeel, T., Shea, T., Priest, M., Abouelliel, A., Sakthikumar, S., Cuomo, C.A., Zeng, Q., Wortman, J., Young, S.K., Earl, A.M., 2014. Pilon: an integrated tool for comprehensive microbial variant detection and genome assembly improvement. PLoS One 9, e112963.

Wood, D.E., Salzberg, S.L., 2014. Kraken: ultrafast metagenomic sequence classification using exact alignments. Genome Biol. 15, R46-2014-15-3-r46.
